# Supplementary material for: Review of some existing QML frameworks and novel hybrid classical–quantum neural networks realising binary classification for the noisy datasets
Source: Sci Rep. 2022 Jul 13;12:11927. doi: 10.1038/s41598-022-14876-6 (PMC9279282; doi:10.1038/s41598-022-14876-6)
Supplement: Supplementary file 1 — Supplementary Information. [file 41598_2022_14876_MOESM1_ESM.pdf]

Supplementary material for:"Binary classifiers for noisy datasets: a comparative study of existing quantum machine learning frameworks and some new approaches"

N. Schetakis[1,5], **D. Aghamalyan**[2,3], **P. Griffin**[3], **and M. Boguslavsky**[4]

[1]School of Electronic and Computer Engineering, Technical University of Crete, Chania, Greece 7310
[2]Centre for Quantum Technologies, National University of Singapore, Singapore 117543
[3]School of Information Systems, Singapore Management University, 81 Victoria Street, Singapore 188065
[4]Tradeteq Ltd, London, UK
[5]ALMA Sistemi Srl, Guidonia (Rome), 00012, Italy

ABSTRACT

Aim of this supplementary section is to show that our FULL HYBRID classifiers perform equally well in high dimensional spaces. Here to gain deeper understanding we demonstrate performance of FULL HYBRID classifiers for 3-dimensional non-convex classification problems. A detailed numerical analysis is done through the extensive benchmarking and following the general approach of the manuscript we also show prediction grids for the chosen dataset.

Benchmarking binary classiffier for 3 dimensional non-convex datasets

In order to gain insights about the performance of our FH classifier architecture in higher dimensions we study classification of 3-dimensional non-convex figure, dataset of which for the different levels of noise is presented in the first rows of Fig 1 and Fig 2. Then we proceed by benchmarking FH classifiers against the QNode and VC. As we can see for this particular dataset, FH:NN/VC-DRC shows the best performance.

We also found it interesting to show (See Fig 2) a 2D projection of the prediction grid for the dataset which has 3 red regions and thus is the hardest non-convex example considered so far. In rows 2-6 we show prediction grids for the VC-DRC, QNode, FH:VC-DRC and FH:NN/VC-DRC, respectively. We see that in the low noise regime FH:VC-DRC/NN and QNode perform the best at the same time, VC-DRC fails to capture upper right corner of that red region. Similar findings hold for the intermediate noise regime, However in the high noise regime FH:VC-DRC/NN and QNode obtain only two regions in red due to too much noise in the dataset, yet VC-DRC is still largely overfitting showing 3 disconnected regions, but guessing their locations wrongly. To summarise, FH classifiers show the maximal resilience to the noise in the dataset and are well suited for non-convex boundary classification problems.

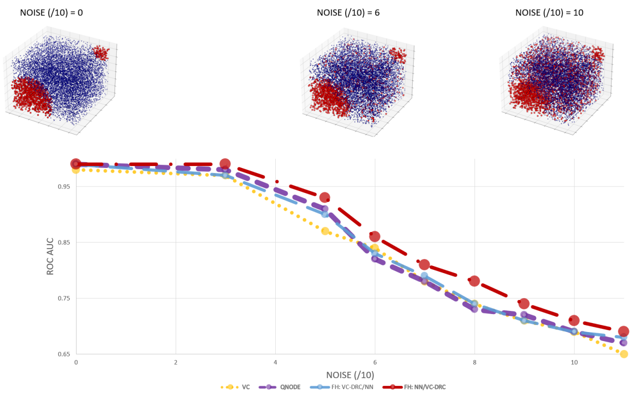

Figure 1. (First Row) Dataset for the 3 dimensional non-convex problem, with increasing noise levels from left to the right (Second row) AUC-ROC curve for the QNode (red dots) , Full-Hybrid (grey dots) and MLPC (orange dots) classifiers for different noise levels.

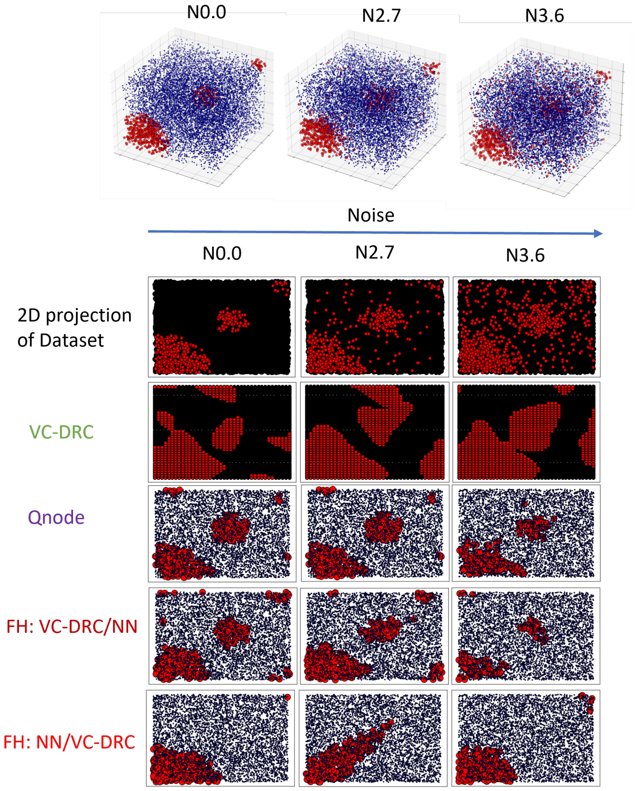

Figure 2. (First row) AUC-ROC curve for classifiers for different noise levels for 3D dataset. (Second row) 2D projection prediction grids for respective classifiers for different noise levels.
